# *Legionella* Metaeffector Exploits Host Proteasome to Temporally Regulate Cognate Effector

**DOI:** 10.1371/journal.ppat.1001216

**Published:** 2010-12-02

**Authors:** Tomoko Kubori, Naoaki Shinzawa, Hirotaka Kanuka, Hiroki Nagai

**Affiliations:** 1 Research Institute for Microbial Diseases, Osaka University, Suita, Osaka, Japan; 2 Graduate School of Frontier Biosciences, Osaka University, Suita, Osaka, Japan; 3 National Research Center for Protozoan Diseases, Obihiro University of Agriculture and Veterinary Medicine, Obihiro, Hokkaido, Japan; The Rockefeller University, United States of America

## Abstract

Pathogen-associated secretion systems translocate numerous effector proteins into eukaryotic host cells to coordinate cellular processes important for infection. Spatiotemporal regulation is therefore important for modulating distinct activities of effectors at different stages of infection. Here we provide the first evidence of “metaeffector,” a designation for an effector protein that regulates the function of another effector within the host cell. *Legionella* LubX protein functions as an E3 ubiquitin ligase that hijacks the host proteasome to specifically target the bacterial effector protein SidH for degradation. Delayed delivery of LubX to the host cytoplasm leads to the shutdown of SidH within the host cells at later stages of infection. This demonstrates a sophisticated level of coevolution between eukaryotic cells and *L. pneumophila* involving an effector that functions as a key regulator to temporally coordinate the function of a cognate effector protein.

## Author Summary

Many bacterial pathogens encode a large array of “effector proteins” that are essential for successful infection. By definition, effector proteins are synthesized in bacteria and transported from bacteria into host cells. Within host cells, effector proteins directly interact with host factors in order to modulate their functions. Effector expression, translocation or activity within host cells must be precisely regulated over infection stages. Here we demonstrate the first example of an effector protein which targets and regulates another effector within host cells: *Legionella* effector protein LubX targets another effector protein SidH to proteasome-mediated protein degradation in the host cells. Expression and delivery of these effector proteins are differentially regulated, which results in LubX-dependent SidH shutdown at late stages of infection. We propose the designation “metaeffector” for this class of bacterial effector protein: an effector that targets and regulates another effector within host cells. Future studies may reveal that metaeffectors which play critical roles in coordinating the functional expression of other effectors spatiotemporally are prevalent among bacterial pathogens.

## Introduction

Many bacterial pathogens encode a large array of “effector proteins,” that manipulate host cellular processes during infection. Effector proteins are translocated from bacteria directly into the cytosol of host cells. This process is mediated by dedicated bacterial protein delivery systems, including the type III and the type IV secretion systems. In some cases, effector proteins delivered into host cells by a bacterium have opposing functions on a single host protein. For example, *Legionella pneumophila* DrrA (SidM) and LepB are effector proteins with opposing effects on the host Rab1 GTPase, with DrrA functioning as a guanine nucleotide exchange factor (GEF) and guanine nucleotide dissociation inhibitor-displacement factor (GDF), and LepB having GTPase-activating protein (GAP) activity[1], [2], [3], [4]. Similarly, the *Salmonella enterica* serovar *typhimurium* effectors SopE and SptP have GEF and GAP activities for the Rho family of GTPases[5], [6], respectively. Although the GEF activity of SopE is dominant in the host cell immediately after infection, degradation of SopE by the host proteasome alters the balance of these effectors, resulting in the GAP activity of SptP to be dominant later in infection [7]. Although differential regulation of gene transcription and post-translational modifications of effectors have also been shown to regulate their activities in host cells[8], [9], details on how these processes are controlled remain largely unknown; other effector-regulating mechanisms probably also exist.

*L. pneumophila* is a gram-negative bacterium ubiquitously found in freshwater environments [10]. When phagocytosed by eukaryotic cells, *L. pneumophila* remodels the *Legionella*-containing phagosome to form a compartment that allows its intracellular replication [11], [12], [13]. As a result, *L. pneumophila* is able to replicate in a wide variety of phagocytic cells, from amoebae to macrophages; human infections can result in a severe pneumonia called Legionnaires' disease [14]. The Dot/Icm type IV secretion system is an essential virulence determinant that translocates *L. pneumophila* effector proteins into host cells during infection [15], [16]. These effector proteins control host cell functions to initiate trafficking of the *L. pneumophila* vacuole, promote host cell survival, modulate innate immune responses, and promote bacterial egress [17]. Although over 100 *L. pneumophila* effector proteins have been identified, the biochemical and cellular functions of most effector proteins remain unknown [17], [18].

**Figure 1:**
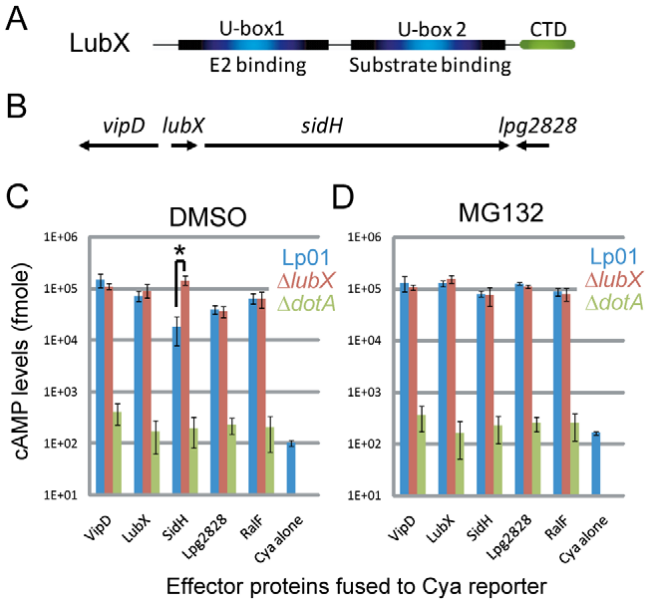
LubX is an ubiquitin ligase that regulates SidH degradation in the host cytosol. (A) Schematic representation of functional regions in LubX. (B) Schematic representation of *lubX* and neighboring genes encoding Dot/Icm type IV secretion system substrates. (C, D) CHO-FcγRII cells were infected with indicated *L. pneumophila* strains carrying a plasmid encoding the indicated Cya fusion proteins or Cya alone under the control of a constitutive promoter. Medium contained solvent alone 0.1% DMSO (panel C) or 10 µM MG132 (panel D) added 30 minutes prior to infection. The *y* axis indicates cAMP levels in cells infected for 8 hours plotted on a logarithmic scale. Data are mean ± SD from three independent samples. *P = 0.005 (*t*-test).

Ubiquitin is a small, well-conserved peptide of 76 amino acids, present in all eukaryotes [19]. Ubiquitination of substrate proteins involves a cascade of reactions. At the last step of the cascade, an E3 ligase recognizes a substrate protein and transfers ubiquitins to the substrate from an E2 conjugating enzyme [20]. Ubiquitinated proteins are subjected to further cellular processes, most notably proteasomal degradation [21]. E3 ligases can be divided into several major families; HECT-type, RING-type, U-box-type and NEL-type [22], [23], [24]. The each family has distinct structural feature, while RING and U-box domains are closely related. Many but not all RING-type E3 ligases work as multi-subunit complexes called SCF complexes containing Skp1, Cullin and F-box proteins. U-box-type E3 ligases contain single U-box domain that serves as an E2-binding site, while *L. pneumophila* effector protein LubX carries two U-box domains, one of which functions as a substrate-binding site (Figure 1A, see below). NEL-type is the most recent addition to E3 ubiquitin ligase families [24]. NEL family is composed of IpaH/SspH family proteins from bacterial pathogens *Shigella flexneri* and *Salmonella enterica* [25], [26], [27] and more than 30 homologous proteins found in bacterial pathogens[24]. Most notably NEL-type E3 ligases seem to be prevalent among bacterial pathogens, whereas no homologous protein has found in eukaryotic cells.

It is well documented that bacterial pathogens exploit the host ubiquitin-proteasome pathway by delivering effectors that function as E3 ubiquitin ligases or as deubiquitinating enzymes [24], [28], [29]. *L. pneumophila* encode one U-box protein (LubX) [30] and several F-box-containing proteins including AnkB/LegAU13/Lpg2144/Lpp2082 [31], [32], [33], [34], [35]. We previously reported that LubX functions as a U-box-type E3 ubiquitin ligase *in vitro* and in host cells [30]. LubX mediates polyubiquitination of a host kinase Clk1, but its consequence remains unknown. Interestingly, the expression and translocation of LubX is induced upon infection and the levels of LubX within host cells come to maximum at later stages of infection, compared to other *L. pneumophila* effectors so far characterized. The gene encoding LubX is in close proximity to genes that encode several other type IV effectors, including VipD [36] and SidH [37] (Figure 1B). Surprisingly, analyses of these effector proteins led us to identify SidH as a target of LubX. LubX acts as a negative temporal regulator of SidH within host cells. This is the first example of the bacterial effector that targets and regulates a cognate effector within the host cells, and we propose the designation “metaeffector” for this class of bacterial effectors.

## Results

### SidH level within host cells is affected by LubX and host proteasome

Translocation of putative effector proteins encoded in vicinity of the *lubX* gene was assessed by measuring cAMP production in the host cytosol generated by an effector containing an amino-terminal fusion to an adenylate cyclase (Cya) domain that is only active in the cytosol of eukaryotic cells[38], [39]. These measurements appear to indicate that translocation of the Cya-SidH fusion protein by wild-type *L. pneumophila* was significantly less than that by an isogenic *lubX* mutant producing the same fusion protein at eight hours post infection (Figure 1C—the asterisk [*], Lp01 *vs*. Δ*lubX*). The difference was the most potent at late stages of infection, while we did not see significant difference at one hour post infection (Figure S1). Translocation of other Cya-tagged effectors such as RalF [40], however, was not affected by the *lubX* mutation (Figure 1C) suggesting that the *lubX* mutation does not have a general effect on type IV secretion. Because LubX has ubiquitin ligase activity, we investigated whether LubX affected SidH translocation through a process requiring host proteasome activity, by treating cells with the proteasome inhibitor MG132. Remarkably, wild type *L. pneumophila* and the *lubX* mutant appeared to translocate Cya-SidH equally in cells treated with the proteasome inhibitor (Figure 1C *vs*. 1D), indicating that inhibition of the host proteasome mimics a bacterial mutant deficient in LubX. These results suggested that LubX-mediated proteasomal degradation of a factor within the host cytosol was required for the reduced levels of Cya-SidH activity. It should be noted though that the Cya fusion assay is not an ideal system to examine the dynamics of intracellular effector levels in infection context, partly because the Cya fusions are under control of a non-authentic constitutive promoter and expressed *in trans*.

### LubX directly binds to SidH

**Figure 2:**
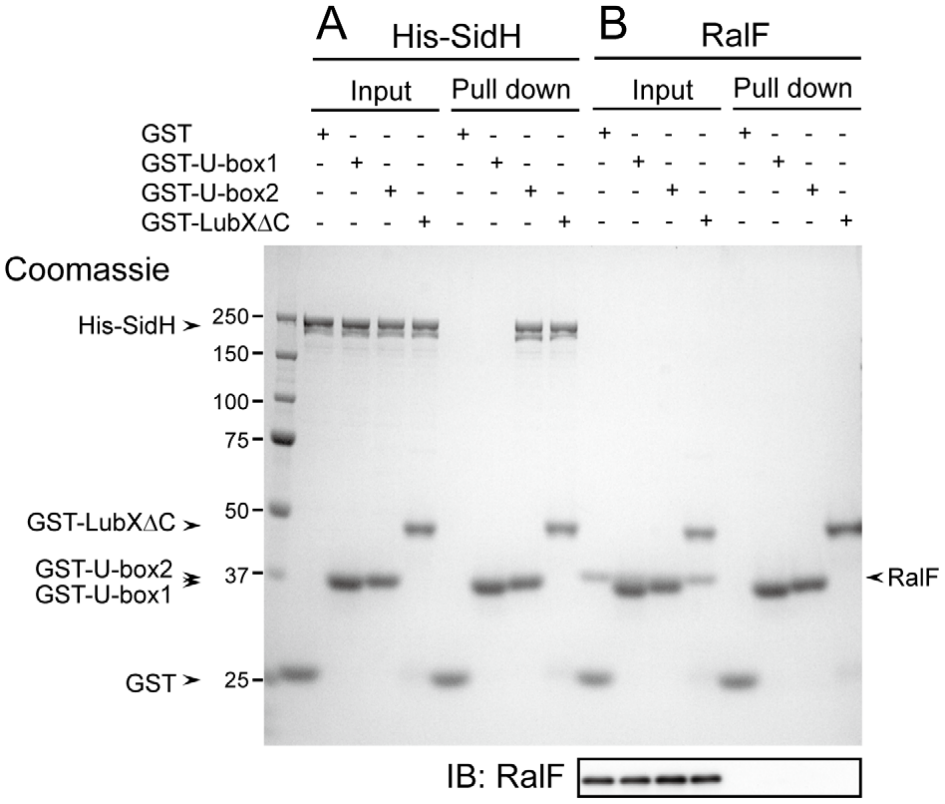
LubX directly binds to SidH but not to RalF. Purified GST or indicated GST fusion proteins (10 µg) was mixed with purified His-SidH (panel A) or RalF (panel B) (5 µg), and protein complexes were isolated by glutathione sepharose. Equivalent amounts of input and pulled down samples were analyzed by 10% SDS-PAGE followed by Coomassie Brilliant Blue staining. Numbers at the left side of the image designate positions of molecular weight markers (in kDa). Because the mobility in the gel of RalF is so close to those of GST-U-box1 and GST-U-box2, the western immunoblotting of the same samples using anti-RalF antibody (IB: RalF) was provided in order to clearly demonstrate that RalF was not pulled-down with any GST derivatives.

Because LubX is able to directly target proteins for degradation, we next examined whether LubX-mediated degradation of SidH in the host cytosol was due to a direct interaction between LubX and SidH. Purified proteins were used to test for direct interactions between SidH and LubX *in vitro* (Figure 2A). His-SidH became bound to the purified GST-LubXΔC, a deletion derivative lacking the C-terminal domain of LubX, but not with GST alone, suggesting interaction between SidH and LubX. The SidH interaction was detected using a fusion protein containing the LubX U-box2 region (GST-U-box2), but not using a fusion protein containing the LubX U-box1 region (GST-U-box1). Another *Legionella* effector RalF became bound neither to GST-LubXΔC nor to GST-U-box2 (Figure 2B), suggesting that LubX U-box2 is a specific protein binding domain. Collectively the U-box2 region of LubX binds specifically and directly to the effector protein SidH.

### SidH is a substrate of LubX E3 ubiquitin ligase

**Figure 3:**
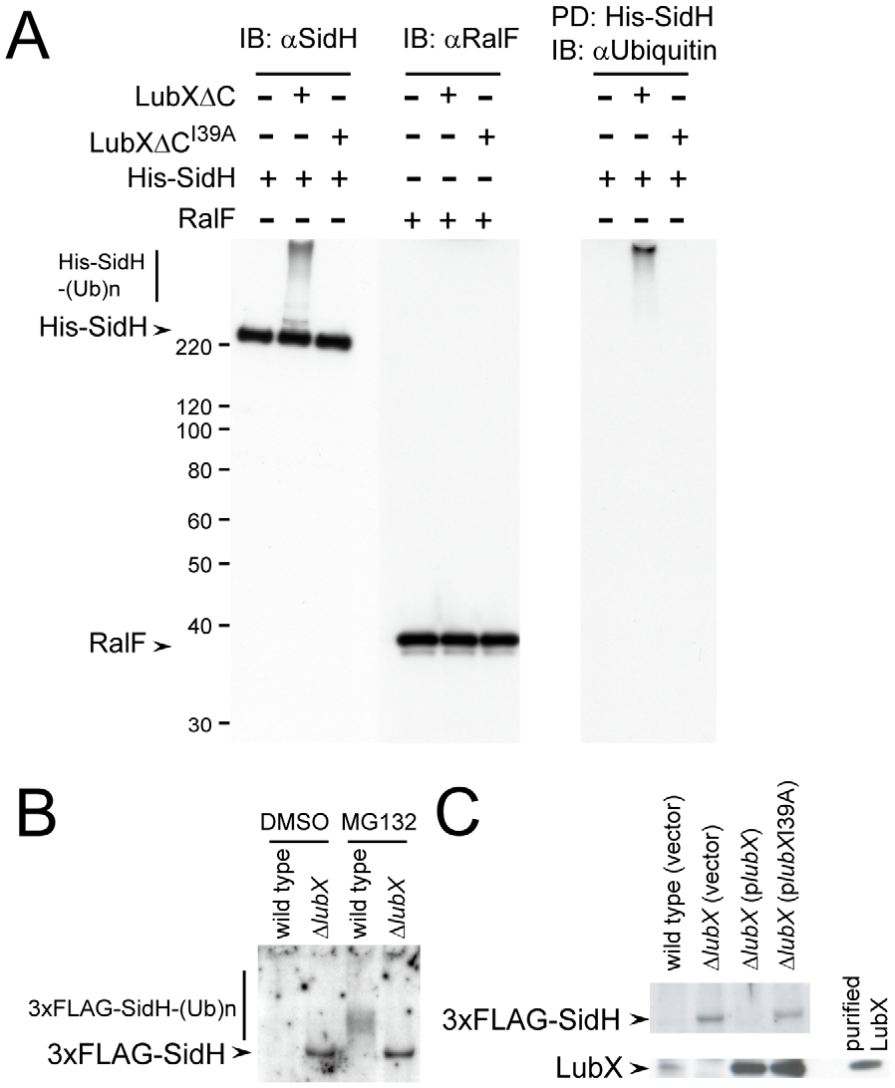
LubX promotes ubiquitination of SidH *in vitro* and within host cell. (A) LubX polyubiquitinates SidH *in vitro*. *In vitro* ubiquitination reactions containing the indicated E3 and substrate proteins were carried out as described in Materials and Methods. The reaction mixtures were analyzed by western immunoblotting using anti-SidH or anti-RalF antibodies (IB: αSidH or IB: αRalF, respectively). In the right panel, His-SidH derivatives in the reaction mixtures were pulled down with nickel resins. Pulled-down materials were analyzed by immunoblotting using anti-Ubiquitin antibody (PD: His-SidH, IB: αUbiquitin). Numbers at the left side of the images designate positions of molecular weight markers (in kDa). (B) LubX-mediated ubiquitination and proteasomal degradation of SidH within the host cells. CHO-FcγRII cells were infected for 8 hours with wild-type or *lubX* deletion strains carrying the triple FLAG-tagged *sidH* gene. DMSO or MG132 was employed as in Figure 1CD. One percent digitonin extracts of the infected cells were subjected to immunoprecipitation with anti-FLAG antibody and the immunoprecipitates were analyzed by immunoblotting using anti-FLAG antibody. (C) The defect in SidH degradation in cells infected with the *lubX* mutant was rescued by producing LubX *in trans*. Indicated strains were used for infection.

An *in vitro* ubiquitination assay was used to determine if LubX binding to SidH could target SidH for ubiquitination. Purified components used were ubiquitin, E1, UbcH5c (E2), LubXΔC or its inactive derivative LubXΔC^I39A^ (E3), His-SidH or another effector protein RalF, and ATP, and the reactions were conducted as described previously[30]. In a functional LubX dependent manner, His-SidH shifted to a very high molecular weight species (Figure 3A, IB: αSidH). Another effector protein RalF was not affected (Figure 3A, IB: αRalF), suggesting the specificity of the reaction. To examine whether the retarded His-SidH species contain ubiquitin, His-SidH was isolated from the reaction mixtures by pull-down using nickel resin and analyzed by western immunibloting using anti-polyubiquitin antibodies (Figure 3A, PD: His-SidH IB: αUbiquitin). The results indicated that the retarded His-SidH species were polyubiquitinated. These results clearly demonstrate that His-SidH is polyubiquitinated by LubXΔC *in vitro*.

### LubX-mediated proteasomal degradation of SidH

To determine whether SidH is polyubiquitinated by LubX in the cytosol of infected host cells, nucleotide sequences encoding an amino-terminal triple-FLAG (3× FLAG) epitope tag were appended to the *sidH* gene on the *L. pneumophila* chromosome. The strain encoding the 3× FLAG-SidH protein expressed SidH at similar levels and the regulation of LubX expression was not affected (Figure S2). Chinese hamster ovary (CHO)-FcγRII cells infected for eight hours with *L. pneumophila* strains producing 3× FLAG-SidH were extracted with a buffer containing 1% digitonin. *L. pneumophila* proteins recovered in the extracts contain proteins translocated into the host cells, but not proteins in the bacterial cells[30], [41]. The 3× FLAG-SidH protein was not detected in cells infected with wild-type *L. pneumophila*, while it was readily detectable in cells infected with the *lubX* mutant (Figure 3B: DMSO, wild type vs Δ*lubX*). Importantly, when host cells were treated with MG132 to inhibit proteasome-mediated degradation, polyubiquitinated 3× FLAG-SidH derivatives were detected from cells infected with *L. pneumophila* producing a functional LubX protein, but only unmodified SidH was detected from cells infected with the isogenic *lubX* mutant (Figure 3B: MG132). The defect in SidH degradation in cells infected with the *lubX* mutant was rescued by producing LubX *in trans* (Figure 3C). Thus LubX is essential for polyubiquitination of SidH in host cells, and polyubiquitinated SidH is degraded by the host proteasome.

### SidH levels are temporally regulated within host cells

**Figure 4:**
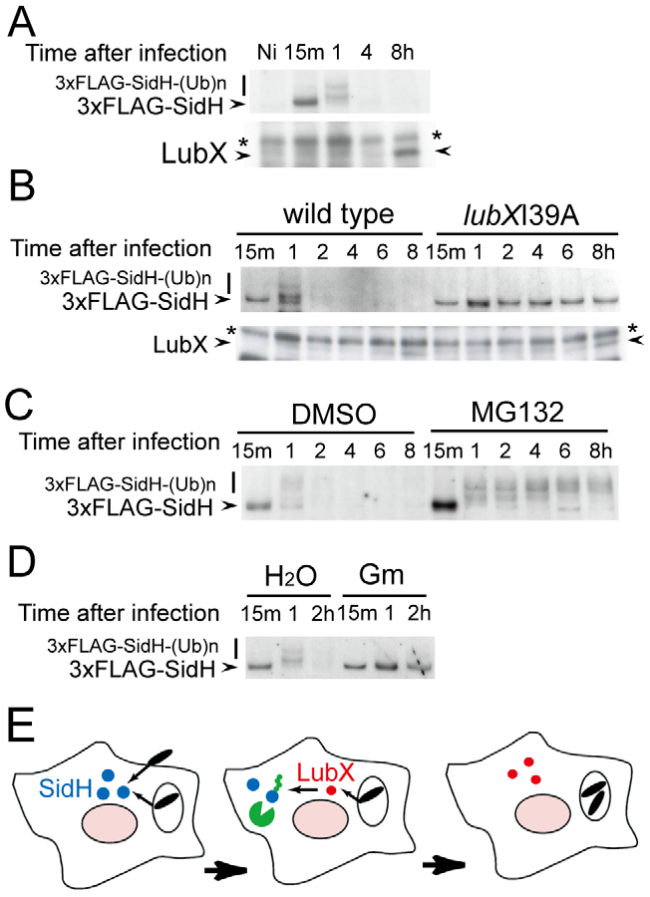
Temporal regulation of SidH is mediated by ubiquitin ligase LubX and host proteasome. (A) Time course of intracellular SidH and LubX levels after infection with *L. pneumophila* strain producing the triple FLAG-tagged SidH. CHO-FcγRII cells were infected and digitonin extracts were prepared at indicated time after infection as in Figure 3B. The Ni lane denotes the non-infected control. Immunoprecipitation and immunoblotting was carried out as in Figure 3B. The asterisks denote a non-specific signal. (B) Shutdown of SidH requires ubiquitin ligase activity of LubX. CHO-FcγRII cells infected with *L. pneumophila* strains carrying wild-type *lubX* gene or *lubX*I39A U-box1 dead mutant were analyzed as in panel A. (C) Shutdown of SidH requires host proteasome. CHO-FcγRII cells were treated with 10 µM MG132 or 0.1% DMSO from 30 minutes before infection with *L. pneumophila*. (D) Shutdown of SidH requires bacterial protein synthesis after infection. *L. pneumophila* was pretreated with 100 µg/ml gentamicin (Gm) or water for 30 minutes and used for infection. (E) Delayed delivery of LubX results in proteasomal degradation of SidH at later stages of infection.

LubX expression by *L. pneumophila* is induced intracellularly. *L. pneumophila* grown extracellularly on laboratory media does not produce LubX, and the levels of LubX increase gradually upon host cell infection, peaking at 10 hours post infection [30] (and Figure S2). In contrast, expression of SidH is induced at the stationary phase of growth in laboratory media, which means SidH levels in the bacterial cell are high when infection is initiated (Figure S2). Accordingly, we hypothesized that SidH levels are regulated intracellularly by the temporal expression and intracellular activities of LubX during infection. SidH levels were measured over time in CHO-FcγRII cells infected with *L. pneumophila* producing 3xFLAG-SidH to test this hypothesis. Full-length 3× FLAG-SidH was detected within the host cells shortly after infection (Figure 4A, 15 m). At one hour post infection, polyubiquitinated 3× FLAG-SidH was detected. Intracellular levels of 3xFLAG-SidH declined over time, and by eight hours post infection, 3× FLAG-SidH was no longer detected. By contrast, LubX levels within the host cells increased over time (Figure 4A), consistent with LubX mediating the intracellular ubiquitination and degradation of SidH. In cells infected with the *L. pneumophila lubX*I39A mutant, similar levels of 3× FLAG-SidH were detected in host cells at all time points and the protein was not polyubiquitinated (Figure 4B: *lubX*I39A), indicating that LubX E3 ubiquitin ligase activity is required for the temporal degradation of SidH. When cells were treated with MG132, we observed the increased levels of polyubiquitinated 3× FLAG-SidH derivatives over time by a process requiring LubX activity (Figure 4C: MG132). Lastly, pretreatment of *L. pneumophila* with the irreversible bacterial translation inhibitor gentamicin abrogated the shutdown of SidH, indicating that intracellular production of LubX was necessary for SidH degradation (Figure 4D: Gm and Figure S3). These results clearly indicate that SidH transiently accumulates within host cells at an early stage of infection, and that the eventual disappearance of SidH in host cells results from LubX-mediated proteasomal degradation (Figure 4E).

### Biological significance of the down regulation of SidH

**Figure 5:**
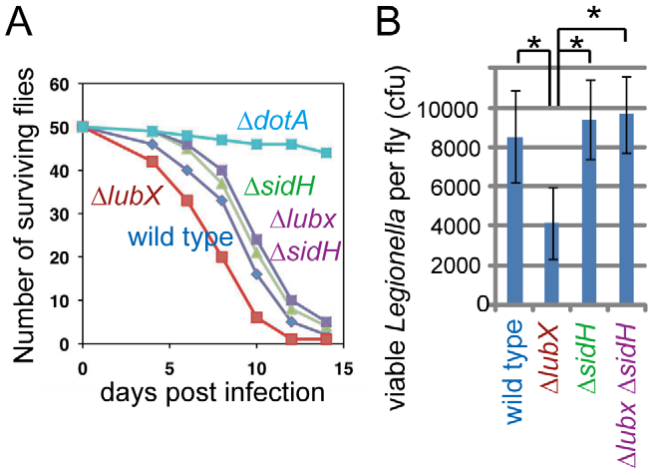
Phenotypes of *lubX*, *sidH* and double knockout strains in fly model. (A) Survival curves of *Drosophila* flies after infection with indicated *L. pneumophila* strains. A representative experiment from at least four independent experiments was shown. Statistics is discussed in the text. (B) Viable bacterial counts in infected flies at 10 days post infection. *P = 0.01 (*t*-test).

The temporal regulation model predicts that the persistence of intracellular SidH led by *lubX* disruption adversely affects *L. pneumophila* fitness in hosts. To address the prediction, we utilized the *Drosophila* infection model [42]. It has been shown that *L. pneumophila* infect and grow to high levels within *Drosophila* cells, and their replication depends on the Dot/Icm type IV secretion system [43]. Correspondingly, although most flies infected with wild-type *Legionella* died within twelve days, ∼80% of flies infected with *Legionella* defective in the Dot/Icm type IV secretion system (Δ*dotA*) survived (Figure 5A). Numbers of the surviving flies infected with the *sidH* mutant were similar to or slightly higher than those infected with the wild-type strain (Figure 5A). The *lubX* mutant consistently showed hyper-lethality to flies compared with the *sidH* mutant or the *lubX sidH* double mutant (Figure 5A, *P*<0.01 for both comparisons). Viable bacterial counts in survived flies infected with the *lubX* mutant were consistently lower than those in flies infected with the wild-type strain (Figure 5B). This apparent defect of replication of the *lubX* mutant in flies was rescued by further introduction of the *sidH* mutant (Figure 5B). Thus, the loss of *lubX* in *sidH* ^+^ *L. pneumophila* gives a disadvantage in multiplication within the model host. It should be noted that the hyper-lethality of the *lubX* mutant did not stem from increased number of viable bacteria (Figure 5B). The *lubX* mutant might be more toxic to a specific type of fly cells (*e.g.* phagocyte) important for survival.

## Discussion

The temporal regulation of SidH mediated by *L. pneumophila* LubX E3 ligase and the host proteasome system illustrates a novel mechanism by which bacterial effectors are regulated. In the previously reported temporal regulation of the induced membrane ruffling by *Salmonella* effectors SopE and SptP, it was shown that these two effectors have distinct susceptibility towards ubiquitin-mediated proteasomal degradation [7]. Importantly, the determinants of the susceptibility are encoded in the effectors themselves. By contrast, the *L. pneumophila* SidH is an intrinsically stable protein within host cells; the effector protein LubX controls SidH instability in the host cytosol by directly targeting this protein for host ubiquitination. Thus LubX represents a *bona fide* metaeffector—a designation for an effector that regulates the function of another effector within the host cell. Discovery of an effector having a regulatory role on another effector provides unique insight into the sophisticated mechanisms that underlie the ability of *L. pneumophila* to coordinate the function of such a large array of effector proteins with diverse activities.

As shown previously [30] and partly in Figure 4 as well, the intracellular level of LubX increases over time and reaches to the maximum at 10–12 hours post infection, which is far late stages compared to the critical time window in which SidH is polyubiquitinated and targeted to proteasomal degradation. It appears that relatively small amount of LubX detected within first couple of hours post infection is sufficient for the polyubiquitination and degradation of SidH (Figure 4). This raised the question on the role of LubX at late stages of infection. We previously reported that host kinase Clk1 is a substrate of LubX [30]. Together with the current findings, one of reasonable explanations would be that LubX has multiple targets within host cells including bacterial SidH and host Clk1.

Accumulating lines of evidence suggest that effector proteins functioning as an E3 ubiquitin ligase are prevalent among many plant and animal bacterial pathogens. Some effector proteins which have been reported to possess E3 ligase activity do not show similarity to eukaryotic E3 ligases at sequence level. AvrPtoB of a plant pathogen *Pseudomonas syringae* shows structural similarity to U-box E3 ligase, whereas they do not share sequence level homology [44]. This suggests that there could be more bacterial E3 ligases than predicted by simple homology search. Structural analyses demonstrated that IpaH/SspH family proteins *Shigella flexneri* IpaH1.4, IpaH3 and *Salmonella enterica* SspH2 show no similarity to eukaryotic E3 ligases and represents a new class of E3 ligase [25], [26], [27]. This class of E3 ligase, called NEL, seems to comprise a large family of bacterial E3 ligases, because more than 30 homologous proteins are found in a subset of bacterial pathogens [24]. Furthermore, there are more than 30 bacterial F-box proteins which are expected to be a key component of SCF E3 ligase complexes [28]. However, the targets of these bacterial E3 ligases remain largely unknown, and all previous studies have aimed to identify host target proteins. SidH is the first example of bacterial targets of the bacterial E3 ligase effector. Future studies without prejudice will reveal that some of bacterial effectors implicated in manipulation of the host ubiquitin system possess regulatory functions as metaeffector that coordinate the expression of effector functions spatiotemporally.

## Materials and Methods

### Bacterial strains, media, plasmids and cell culture

All *Legionella* strains used in this study were derivatives of *L. pneumophila* strain Lp01 [45] and were grown on charcoal-yeast extract (CYE) plates or in ACES-buffered yeast extract (AYE) broth as described previously[46]. The strains defective in *lubX* and *dotA* genes have been previously described [30], [47]. *Legionella* strains defective in *sidH* and *lubX-sidH* genes as well as *Legionella* strains carrying 3xFLAG-*sidH* or the *lubX* I39A mutation were constructed by allelic exchange[47]. *E. coli* strain BL21 (strain B *lon ompT*) was used as an expression host for protein purification. Plasmids used in this study and details of plasmid construction are provided in the Tables S1 and S2. Chinese hamster ovary (CHO)-FcγRII cells were cultured at 37°C in 5% CO_2_ in α-MEM, supplemented with heat-inactivated 10% FBS, as described[48].

### Antibodies

Custom made anti-serum against SidH peptide CQNIKGPEPVATPMETPE (SidH 2196–2212) was purchased from MBL. Antibodies were purified from the anti-serum by affinity chromatography using peptide-conjugated SulfoLink resins (Pierce). The affinity-purified rabbit polyclonal antibodies against RalF, LubX and GroEL were described previously [30], [40]. Mouse monoclonal antibody against polyubiquitin (clone FK1) was purchased from BIOMOL. Monoclonal antibody against the FLAG tag (M2) was purchased from Sigma-Aldrich.

### Cya reporter assay

Translocation of Cya-fused proteins into CHO-FcγRII cells after infection with *Legionella* was assayed as described previously [30], [38] with minor modifications. Briefly, CHO-FcγRII cells were replated in 24-well plates, and challenged by *Legionella* strains expressing Cya fusions at a multiplicity of infection (moi) of 30 in the presence of opsonizing antibody (1∶3000 dilution). Eight hours later, infected cells were lysed in 500 µl of lysis reagent 1B provided from a cAMP Biotrak EIA System (GE Healthcare, RPN2251); cAMP levels were determined according to manufacturer's instructions.

### Protein purification

Purification of LubXΔC, RalF and GST fusion proteins has been described previously [30], [40]. For purification of His-SidH, BL21 cell pellets from a 2-liter culture expressing His-SidH were suspended with 80 ml PBS containing Complete Protease Inhibitor Cocktail (Roche) and 20 mg lysozyme (Wako Chemical). After incubation with stirring for 30 minutes at 4°C, the lysozyme-treated cells were lysed by sonication. After centrifugation (16,000× g for 20 minutes) to remove unsolubilized materials, the supernatant fraction was mixed with ammonium sulfate (final 40% saturation) and incubated for 30 minutes at 4°C. After centrifugation to remove precipitates, the supernatant fraction was mixed with ammonium sulfate (final 60% saturation) and incubated for 30 minutes further at 4°C. After centrifugation, the precipitates were dissolved in 20 ml PBS containing Complete Protease Inhibitor Cocktail (Roche). This solution was dialyzed against PBS to remove residual ammonium sulfate. After centrifugation to remove insoluble materials, the supernatant fraction was mixed with 6 ml (bed volume) HIS-Select Resin (Sigma-Aldrich) and incubated for 30 minutes at 4°C. The resins were washed 5 times with PBS containing 5 mM imidazole, and the bound proteins were eluted with 6 ml PBS containing 100 mM imidazole. The elution step was repeated once more, and the resulting two eluate fractions were pooled. The pooled fraction was mixed with half its volume of 20 mM Tris HCl, pH 7.5 to reduce salt concentration (final 0.1 M NaCl). The resulting solution was applied to a MonoQ 5/50GL chromatography column (GE healthcare). After elution by NaCl gradient (0.1 M to 0.5 M in 20 mM Tris HCl, pH. 7.5), the peak fractions were further subjected to a Superose 6 10/300GL column (GE healthcare) equilibrated with 20 mM Tris HCl pH 7.5, 150 mM NaCl. The peak fractions were pooled and concentrated using a Microcon device (Millipore).

### GST pull-down assays

For a GST pull-down using purified proteins, GST, GST-LubXΔC, GST-U-box1 or GST-U-box 2 (10 µg) were mixed with 5 µg of His-SidH or RalF in 500 µl PBS containing 1 mM EDTA, 1 mM DTT, and 1% (w/v) Triton X-100. The resulting solutions were mixed with 25 µl of a 50% suspension of Glutathione-Sepharose and incubated for three hours with gentle rotation at 4°C. Unbound proteins were removed by centrifugation, and resins were washed four times with the same buffer, and once with a buffer omitting TritonX-100. GST and interacting proteins were eluted with 50 µl of SDS sample buffer containing reducing agent.

### Ubiquitin ligase assays

The *in vitro* ubiquitin polymerization assay was performed essentially as described [30] with a couple of modifications; 300 nM E3 enzyme (LubX derivatives) were employed; where indicated, 240 nM of purified His-SidH or RalF was included. Where indicated, His-SidH derivatives were pulled down with His-Select resin (Sigma-Aldrich) and washed in the presence of 2.5 mM imidazole to suppress nonspecific interaction between reaction component proteins and the resin. Pulled-down materials were eluted with 250 mM imidazole. Samples were subjected to 10% SDS-PAGE and analyzed by immunoblotting using antibodies against SidH, RalF or polyubiquitin.

### Fractionation of infected cells

CHO-FcγRII cells were replated in a 6-well culture dish, and challenged by *Legionella* strains at a moi of 30 in the presence of opsonizing antibody (1∶3000 dilution). One hour after infection, the cells were washed three times with PBS (pre-warmed to 37°C) to remove non-internalized bacteria, then further incubated in cell culture medium. When indicated, 10 µM of MG132 (Calbiochem) or equivalent amount of the solvent DMSO was added to the cell medium 30 min prior to infection as well as to the replacing medium. At the indicated time points, the cells were washed three times with cold PBS and lysed in 150 µl of PBS containing 1% (w/v) of digitonin (Calbiochem), 10 mM of N-ethylmaleimide (Sigma) to prevent deubiquitination, and protease inhibitor cocktail (1∶100 dilution, Sigma-Aldrich). The cells were scraped off, collected into microfuge tubes and centrifuged at 16,000× g for 10 min at 4°C to separate the digitonin-soluble fraction containing translocated proteins from the digitonin-insoluble fraction containing internalized bacteria. The digitonin-soluble fractions were filtrated through a 0.45 µm filter unit (Millex-HV, Millipore). Immunoprecipitates with anti-FLAG or anti-LubX antibodies, extracted using nProteinA Separose (GE Healthcare), were analyzed by SDS-PAGE followed by immunoblotting using anti-FLAG or anti-LubX antibodies, respectively.

### Infection to fruit flies and colony forming assay

Five to seven days olds *yw* male *Drosophila melanogaster* flies were used for infection experiments. Before injection, the bacteria-containing medium was adjusted to 0.1 OD using Gene Quant *pro* (Amersham) with distilled water. Flies were anesthetized with CO_2_ and injected with each strain of bacteria in 65 nl of water (approximately 500 colony-forming units). Injection was carried out by using an individually calibrated pulled glass needle attached to IM-300 microinjector (Narishige). Flies were always injected in the abdomen, close to the junction with thorax and just ventral to the junction between the ventral and dorsal cuticles. After injection, flies were transferred to fresh vials once a week. For colony-forming assay, 10 days after bacterial injection flies were homogenized in 10 mM MgSO_4_ solution and the diluted series of the homogenized samples were plated on CYE media containing 100 µg/ml streptomycin.

## Supporting Information

Table S1 Plasmids used in this study.
(0.05 MB DOC)

Table S2 Details of plasmid construction.
(0.03 MB DOC)

Figure S1 Time course of Cya activity in CHO-FcγRII cells infected with *Legionella* strains producing Cya-SidH. Infection was carried out as in Figure 1CD, with a modification of using lower multiplicity of infection (moi  = 3). At indicated time points, samples were prepared and analyzed as in Figure 1CD.
(0.14 MB TIF)

Figure S2 Triple-FLAG tagging to SidH encoded on the chromosome did not affect the levels of SidH expressed in *Legionella* grown in laboratory media. Wild type (Lp01) or its isogenic strain carrying triple-FLAG tag insertion in the sidH gene on chromosome (3xFLAG-*sidH*) were grown in AYE liquid medium or on CYE solid medium. Whole cell lysates were prepared from *Legionella* grown as indicated, and were analyzed by immunoblotting using antibodies against the indicated proteins. For AYE-grown *Legionella*, samples were taken at the exponential phase (EP) or post-exponential phase, 12 hours or 18 hours after inoculation (PE). For CYE-grown *Legionella*, samples were taken from a heavy patch after 48 hours of incubation.
(0.25 MB TIF)

Figure S3 Gentamycin (Gm) pretreatment of *L. pneumophila* eliminates LubX synthesis after infection. LubX level was monitored at 8 hours post infection in lysates of CHO-FcγRII cells infected with wild-type *L. pneumophila* pretreated with gentamicin or distilled water. Asterisk denotes nonspecific signal and serves as a loading control.
(0.13 MB TIF)
