# Supplementary material for: Legionella Metaeffector Exploits Host Proteasome to Temporally Regulate Cognate Effector
Source: PLoS Pathog. 2010 Dec 2;6(12):e1001216. doi: 10.1371/journal.ppat.1001216 (PMC2996335; doi:10.1371/journal.ppat.1001216)
Supplement: Table S1 — Plasmids used in this study. (0.05 MB DOC) [file ppat.1001216.s001.doc]

**Table S1. Plasmids used in this study.**

**Plasmids Properties References**

pmEGFP pEGFP-C2 L221K [1] [2]

pNH1249 pmEGFP harboring the full-length *sidH* gene This study

pMMB207 Cloning vector derived from RSF1010 [3]

pMMB207-M45NT Cloning vector for N-terminal M45-tagging [4]

pNH1206 pMMB207-M45NT encoding LubX This study

pNH1208 pMMB207-M45NT encoding LubX I39A This sutudy

pcya-ralF pMMB207-M45NT encoding cya-ralF [5]

pNH1301 pcya- lubX [2]

pNH1255 pcya-sidH This study

pNH1256 pcya-vipD This study

pNH1258 pcya-lpg2828 This study

pSB1975 pGEX-4T3 based expression vector C.E. Stebbins

pNH1044 pSB1975 encoding GST-LubX This study

pNH1045 pSB1975 encoding GST-LubX I39A This study

pNH1101 pSB1975 encoding GST-LubXΔC [2]

pNH1344 pSB1975 encoding GST-Ubox1 This study

pNH1229 pSB1975 encoding GST-Ubox2 [2]

pGST-ralF pGEX-KG encoding GST-RalF [6]

pET15b *E. coli* expression vector Novagen

pNH1253 pET15b encoding His-sidH This study

pSR47S Gene replacement vector [7]

p3XFLAG-CMV-10 used as 3xFLAG donor Sigma-Aldrich

pNH1262 pSR47S carrying *sidH* upstream region This study

pNH1263 p3XFLAG-CMV-10 carrying 3xFLAG-*sidH′* This study

pNH1264 pSR47S carrying 3xFLAG-*sidH* allele This study

pNH1063 pmEGFP encoding LubX I39A [2]

pNH1271 pNH1063 carrying a *lubX* upstream region This study

pNH1272 pSR47S carrying *lubX* I39A allele This study

pNH1018 pSR47S carrying Δ*sidH* allele This study

pNH1022 pSR47S carrying Δ*lubX*Δ*sidH* allele This study

**References to Table S1**

1. Snapp EL, Hegde RS, Francolini M, Lombardo F, Colombo S, et al. (2003) Formation of stacked ER cisternae by low affinity protein interactions. J Cell Biol 163: 257-269.

2. Kubori T, Hyakutake A, Nagai H (2008) *Legionella* translocates an E3 ubiquitin ligase that has multiple U-boxes with distinct functions. Mol Microbiol 67: 1307-1319.

3. Morales VM, Backman A, Bagdasarian M (1991) A series of wide-host-range low-copy-number vectors that allow direct screening for recombinants. Gene 97: 39-47.

4. Coers J, Kagan JC, Matthews M, Nagai H, Zuckman DM, et al. (2000) Identification of icm protein complexes that play distinct roles in the biogenesis of an organelle permissive for *Legionella pneumophila* intracellular growth. Mol Microbiol 38: 719-736.

5. Nagai H, Cambronne ED, Kagan JC, Amor JC, Kahn RA, et al. (2005) A C-terminal translocation signal required for Dot/Icm-dependent delivery of the *Legionella* RalF protein to host cells. Proc Natl Acad Sci U S A 102: 826-831.

6. Nagai H, Kagan JC, Zhu J, Kahn RA, Roy CR (2002) A Bacterial Guanine Nucleotide Exchange Factor Activates ARF on *Legionella* Phagosomes. Science 295: 679-682.

7. Merriam JJ, Mathur R, Maxfield-Boumil R, Isberg RR (1997) Analysis of the *Legionella pneumophila* *fliI* gene: intracellular growth of a defined mutant defective for flagellum biosynthesis. Infect Immun 65: 2497-2501.
