# Supplementary material for: Legionella Metaeffector Exploits Host Proteasome to Temporally Regulate Cognate Effector
Source: PLoS Pathog. 2010 Dec 2;6(12):e1001216. doi: 10.1371/journal.ppat.1001216 (PMC2996335; doi:10.1371/journal.ppat.1001216)
Supplement: Table S2 — Details of plasmid construction. (0.03 MB DOC) [file ppat.1001216.s002.doc]

**Table S2. Details of plasmid construction**

Plasmids Vectors Primers Restriction enzyme sites Restriction Enzymes

No. Sequence

pNH1249 pmEGFP #574 gcgAGATCTctgaattcatgaaaagaaccattgaaacc BglII

#550 gggatcctctagatgattAGATCTtctcttctg BglII

#551 ggagctcgGAATTCcttaagaagatgc EcoRI

#575 gggatccGTCGACtctagattagaatctggtaatattggc SalI

(1.2kb N-terminal and 1.1kb C-terminal fragments of *sidH* were obtained by PCR and the internal region was from shotgun cloning from genomic DNA. These were serially subcloned to the vector.)

pNH1206 pMMB207NT #27 cgGGATCCttatggcgacacgaaatccttttg BamHI

#28 gcgCTGCAGttagtacgtcatatcagatg PstI

pNH1208 pMMB207NT #27 cgGGATCCttatggcgacacgaaatccttttg BamHI

#28 gcgCTGCAGttagtacgtcatatcagatg PstI

(pNH1045 was used as a PCR template.)

pNH1255 pcya-ralF subcloned from pNH1249 BglII/BamHI

XbaI

pNH1256 pcya-ralF #581 cGGATCCttatgacaaaaagccgtaaattaaaaag BamHI

#582 gcgcgAAGCTTaatggccgccaaatgtgg HindIII

pNH1258 pcya-ralF #585 cGGATCCttatgaaaattagtgaattaaaaaaac BamHI

#586 gcgCTGCAGttaatttcttagtaaaggatag PstI

pNH1044 pSB1975 #337 ggaattcAAGCTTatggcgacacgaaatcc HindIII

#338 gcCTCGAGccatggttagtacgtcatatcag XhoI

pNH1045 pNH1044 #345 atgctgacttgccctgctgacagcggatttcta

#346 tagaaatccgctgtcagcagggcaagtcagcat

(This plasmid was constructed by site-directed mutagenesis of pNH1044. Site-directed mutagenesis was carried out using Quick Change Kit (Stratagene) and indicated primers according to the manufacture’s instruction.)

pNH1344 pSB1975 #337 ggaattcAAGCTTatggcgacacgaaatcc HindIII

#416 gcCTCGAGccatggttacgtttgtgtctcaacaaatc XhoI

pNH1253 pET15b subcloned from pNH1249 EcoRI/NdeI

SalI/XhoI

(EcoRI and NdeI sites were filled in prior to ligation)

pNH1262 pSR47S #602 gcGAGCTCgaattcttgatgaactcta tcaacag SacI

#603 gcgcgCCATGGcacatcaaccagcaaatgaac NcoI

(cloning to the pSR47S derived another construct having SacI/NcoI sites)

pNH1263 p3XFLAG-CMV-10

#604 gcgcgAAGCTTcggatccttatgaaaagaaccattgaaacc HIndIII

#605 gcTCTAGAgcgatcatggcagtgtacc XbaI

pNH1264 pNH1262 subcloned from pNH1263

pNH1271 pNH1063 #662 gcGCTAGCggctttttgtcataatttc NheI

#663 ctCTTAAGtatttacttttatgatc AflII

pNH1272 pSR47S #664 gcGAGCTCggctttttgtcataatttc SacI

#659 gcTCTAGAttagtacgtcatatcagatg XbaI

(pNH1271 was used as a PCR template)

pNH1018 pSR47S #111 gcTCTAGAgaactctatcaacagataaaag XbaI

#101 ttaTCTAGAcatgccacatcaaccagcaaatg XbaI

#102 atgTCTAGAtaattcgaattagttttg XbaI

#103 gcGAGCTCgtcttttgtctggacaaatac SacI

pNH1022 pSR47S #110 gcTCTAGAggctttttgtcataatttc XbaI

#98 ttaTCTAGAcatttctattctatacccc XbaI

#102 atgTCTAGAtaattcgaattagttttg XbaI

#103 gcGAGCTCgtcttttgtctggacaaatac SacI

Unless otherwise indicated, plasmids were constructed by ligation of PCR-amplified fragments using Lp01 genomic DNA as templates into the vectors indicated. The table provides the primers used for PCR amplification of insert DNA, the recipient vector, and the restriction enzymes used for DNA digestion. Restriction enzyme sites are shown in uppercase.
