# Supplementary figures and images for: Legionella Metaeffector Exploits Host Proteasome to Temporally Regulate Cognate Effector
Source: PLoS Pathog. 2010 Dec 2;6(12):e1001216. doi: 10.1371/journal.ppat.1001216 (PMC2996335; doi:10.1371/journal.ppat.1001216)

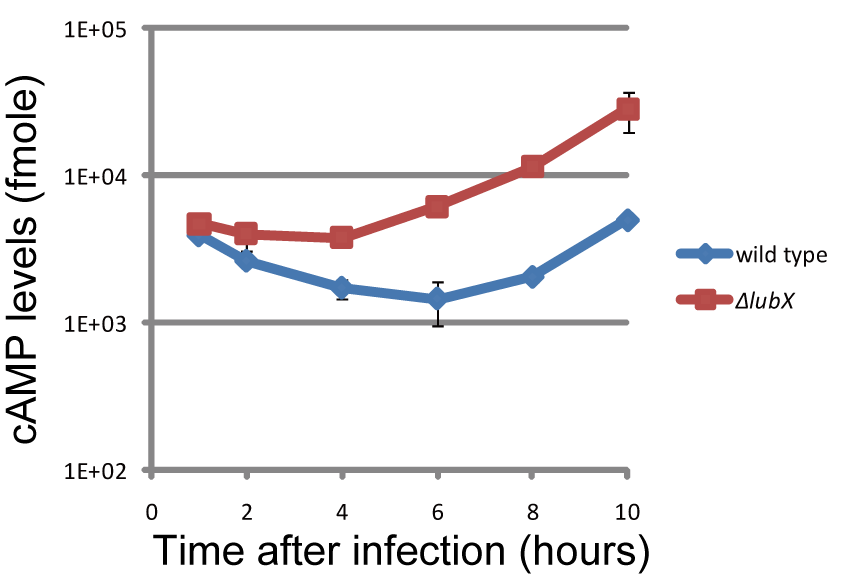

Supplement: Figure S1 — Time course of Cya activity in CHO-FcγRII cells infected with Legionella strains producing Cya-SidH. Infection was carried out as in Figure 1CD, with a modification of using lower multiplicity of infection (moi = 3). At indicated time points, samples were prepared and analyzed as in Figure 1CD. (0.14 MB TIF) [file ppat.1001216.s003.tif]

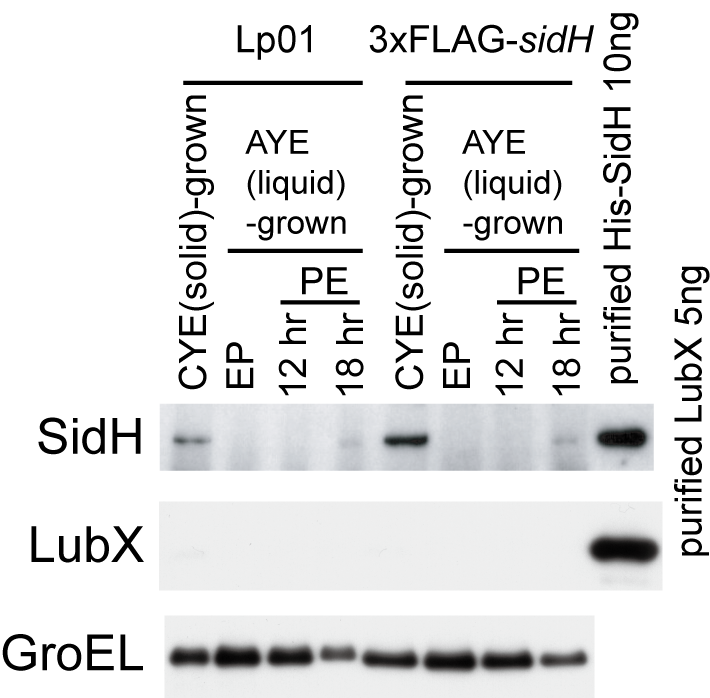

Supplement: Figure S2 — Triple-FLAG tagging to SidH encoded on the chromosome did not affect the levels of SidH expressed in Legionella grown in laboratory media. Wild type (Lp01) or its isogenic strain carrying triple-FLAG tag insertion in the sidH gene on chromosome (3xFLAG-sidH) were grown in AYE liquid medium or on CYE solid medium. Whole cell lysates were prepared from Legionella grown as indicated, and were analyzed by immunoblotting using antibodies against the indicated proteins. For AYE-grown Legionella, samples were taken at the exponential phase (EP) or post-exponential phase, 12 hours or 18 hours after inoculation (PE). For CYE-grown Legionella, samples were taken from a heavy patch after 48 hours of incubation. (0.25 MB TIF) [file ppat.1001216.s004.tif]

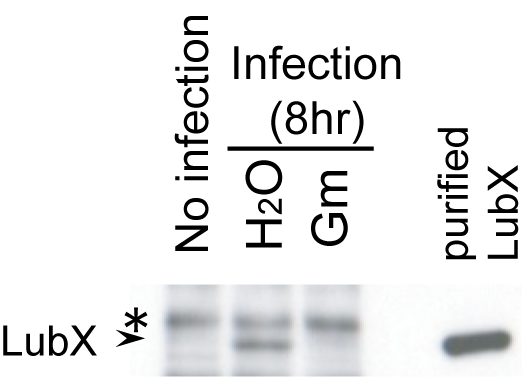

Supplement: Figure S3 — Gentamycin (Gm) pretreatment of L. pneumophila eliminates LubX synthesis after infection. LubX level was monitored at 8 hours post infection in lysates of CHO-FcγRII cells infected with wild-type L. pneumophila pretreated with gentamicin or distilled water. Asterisk denotes nonspecific signal and serves as a loading control. (0.13 MB TIF) [file ppat.1001216.s005.tif]
